# Plant-Based Diet Indices and Their Association with Frailty in Older Adults: A CLHLS-Based Cohort Study

**DOI:** 10.3390/nu15245120

**Published:** 2023-12-15

**Authors:** Ran Qi, Yun Yang, Baihe Sheng, Huiping Li, Xinyu Zhang

**Affiliations:** School of Public Health, Tianjin Medical University, Tianjin 300070, China; qiran@tmu.edu.cn (R.Q.); yunyang@tmu.edu.cn (Y.Y.); shengbaihe@tmu.edu.cn (B.S.); lihuiping1222@163.com (H.L.)

**Keywords:** Chinese longitudinal healthy longevity survey, older adults, plant-based diet indices, frailty

## Abstract

Within the realm of aging, the nexus between diet and health has garnered considerable attention. However, only select studies have amalgamated insights into the correlation between plant and animal food consumption and frailty. Our aim was to appraise the connections between the overall plant-based diet index (PDI), healthful plant-based diet index (hPDI), and unhealthful plant-based diet index (uPDI) and frailty in the elderly, utilizing data from the Chinese Longitudinal Healthy Longevity Survey (CLHLS). This cohort study drew upon CLHLS data spanning from 2008 to 2018. The PDI, hPDI, and uPDI were gauged using a simplified food frequency questionnaire (FFQ). A frailty index, encompassing 35 variables across major health domains, was formulated. Cox proportional hazard models were employed to scrutinize the associations between the three plant-based dietary indices and frailty in older adults, including an exploration of gender disparities in these associations. A cohort of 2883 study participants was encompassed, with 1987 (68.9%) observed to be either frail or in the pre-frail stage. The Cox model with penalized spline exhibited linear associations of PDI, hPDI, and uPDI with the frailty index. Following covariate adjustments, it was discerned that older adults situated in the highest quartiles of PDI (HR = 0.86, 95% CI: 0.77–0.95) and hPDI (HR = 0.83, 95% CI: 0.74–0.93) experienced a 14% and 17% diminished risk of frailty compared to those in the lowest quartiles of PDI and hPDI, respectively. Conversely, when contrasted with those in the lowest quartile of uPDI, older adults adhering to the highest tertile of uPDI exhibited a 21% elevated risk of frailty (HR = 1.21, 95% CI: 1.08–1.36), with both associations achieving statistical significance (*p* < 0.01). Moreover, additional subgroup analyses revealed that the protective effects of PDI and hPDI against frailty and the deleterious effects of uPDI were more conspicuous in men compared to women. To forestall or decelerate the progression of frailty in the elderly, tailored dietary interventions are imperative, particularly targeting male seniors.

## 1. Introduction

Frailty, defined as a state of vulnerability marked by cumulative decline in physiological systems and a diminished resistance to stressors [1], constitutes an escalating global health concern, notably prevalent among the elderly. Previous data have indicated that the prevalence of frailty and pre-frailty in the elderly reached 24% and 49%, respectively [2]. Projections suggest that the prevalence of frailty will continue to escalate in the context of the ongoing global ageing trend [3]. Frailty typically manifests as a general decline in various systems and organs, characterized by compromised resistance and responsiveness to stressors [4]. This condition not only gives rise to heightened clinical symptoms and adverse outcomes, including mobility disability, falls, cardiovascular diseases, and cognitive decline, but also elevates the risk of hospitalization and mortality in the elderly [5,6,7,8]. The public health implications associated with frailty exert substantial pressure on healthcare systems worldwide, and the health of older individuals is increasingly under scrutiny. While frailty is dynamic and preventable, effective interventions become challenging when it progresses to an advanced stage before death [9]. Therefore, adopting proactive strategies to prevent frailty or identify and delay its onset in the early stages is pivotal for enhancing the quality of life and well-being of the elderly in their later years.

The etiology of frailty in the elderly is multifactorial, influenced by environmental, behavioural, and nutritional factors [10,11,12,13]. As a modifiable aspect of lifestyle, diet has garnered increasing attention for its role in promoting human health. It has been demonstrated that habitual tea consumption and a high intake of fruits and vegetables are efficacious in preventing frailty development [14,15,16], whereas habitual red meat intake is associated with an elevated risk of frailty [17]. However, assessing only individual foods and the relative limitations of nutrients can be mitigated by the plant-based diet index, which incorporates multiple food groups and comprehensively considers their health benefits [18,19,20]. By encompassing common food groups in the population and categorizing them into healthful plant-based foods, unhealthful plant-based foods, and animal-based foods based on their health benefits, the plant-based diet index offers greater flexibility and enhanced population promotion compared to the conventional “Mediterranean diet” and “Western diet” [21,22,23].

Only three studies have investigated the correlation between a plant-based dietary index and frailty. Two of these studies were conducted among female nurses [24] and community-dwelling older adults [25], resulting in limitations related to the generalization of findings to broader populations. Another study, exclusive to Chinese elderly participants, solely examined the association between the Plant-based diet index (PDI) and frailty [26]. Furthermore, all these studies employed the physical frailty phenotype as the basis for defining frailty, a categorization that does not directly correlate with a specific disease or significant disability. This phenotype overlooks cognitive and emotional aspects and proves inadequate for characterizing frailty linked to known comorbidities [27]. Divergent from the frailty phenotype, the deficit accumulation model incorporates numerous candidate factors, encompassing all major health domains (e.g., cognition, emotion, chronic disease, and cognitive function), and its multidimensional advantage facilitates the understanding of frailty across various pathogenic pathways. The frailty index employed in this study is grounded in the deficit accumulation model, offering a pragmatic measure of frailty. This study sought to investigate the associations between three plant-based dietary indices and frailty in individuals aged 65 and above in China, utilizing nationally representative cohort data. Additionally, the study aimed to explore gender-specific differences in these associations, providing a scientific reference for older individuals to adapt their dietary habits and forestall frailty.

## 2. Objects and Methods

### 2.1. Study Population

The Chinese Longitudinal Healthy Longevity Survey (CLHLS) commenced in 1998 with the aim of conducting a nationally representative survey of Chinese individuals aged 65 years and above. The primary objective of the CLHLS is to scrutinize the correlation between common health-related factors and health outcomes in the elderly, providing a valuable reference for promoting healthy ageing. The survey encompassed 23 provinces, municipalities, and autonomous regions, covering approximately 85% of China’s population. Information gathered by the project included fundamental characteristics, socioeconomic features, behavioural habits, dietary status, and physical health status of the elderly. More detailed descriptions of the CLHLS project can be found elsewhere [28,29,30]. The CLHLS received ethical approval from the Biomedical Ethics Committee of Peking University, China (IRB00001052–13074).

**Figure 1:**
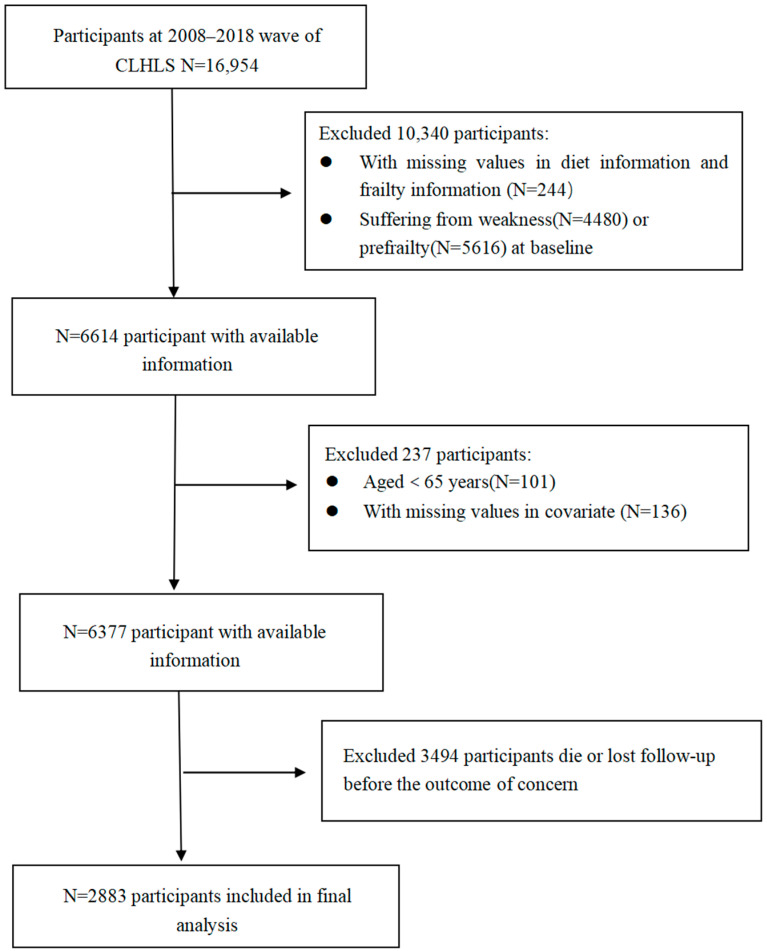
Flowchart of Participant Screening Flowchart.

This study utilised data extracted from the 2008–2018 CLHLS. A total of 16,954 participants aged 65 years and older were enrolled for this follow-up analysis. Exclusions were applied to those with missing dietary information, frailty details, and covariates at baseline and during follow up. Additionally, older adults presenting with frailty at baseline or in the pre-frailty stage were excluded, retaining only those without predisposition to frailty at baseline. Furthermore, individuals who experienced mortality or were lost to follow up without an outcome of interest during the follow-up period were also excluded. The analysis ultimately included a final cohort of 2883 participants. A comprehensive depiction of participant enrolment and exclusion processes is presented in Figure 1.

### 2.2. Calculation of Plant-Based Diet Indices

The dietary information of the subjects was acquired through a simplified food frequency questionnaire (FFQ). A total of 16 common food groups in the daily Chinese diet were encompassed, categorised into three groups based on the nature of the food: healthful plant-based foods (whole grains, vegetable oils, fruits, vegetables, soy products, garlic, nuts, and tea), unhealthful plant-based foods (refined grains, preserved vegetables, and sugar), and animal-based foods (animal fats, meat, fish and other aquatic products, eggs, and dairy products). From the self-reported dietary frequency, we computed three plant-based diet indices: an overall plant-based diet index (PDI), a healthful plant-based diet index (hPDI), and an unhealthful plant-based diet index (uPDI). The consumption of each food was taken into account in the calculation of the three plant-based diet indices. Drawing from prior research [18,31,32,33], each food item was assigned a score between 1 and 5, with subtle variations in emphasis for the three plant-based diet indices. Among them, PDI focuses on the high consumption of plant food and the low consumption of animal food in the study population. hPDI highlights the high consumption of healthy plant foods and the low consumption of unhealthy plant foods; uPDI, in contrast to hPDI, focuses on high consumption of unhealthy plant foods and low consumption of healthy plant foods. For the PDI, scores were allocated based on the frequency of consumption, favouring healthful plant-based diets over unhealthful ones (5 points for the most frequent consumption, and 1 point for rarely or never consumption). In the case of hPDI, a higher frequency of consumption of healthful plant-based diets garnered a higher score (5 points for the most frequent consumption, and 1 point for rarely or never consumption), while unhealthful plant-based and animal-based diets were inversely scored (1 point for the most frequent consumption, and 5 points for rarely or never consumption). The uPDI assigned a higher score for a more frequent consumption of an unhealthful plant-based diet (5 points for the most frequent consumption, and 1 point for rarely or never consumption), with an inverse scoring for healthful plant-based diets and animal-based diets (1 point for the most frequent consumption, and 5 points for rarely or never consumption). For animal foods, 1 point is given for most frequent consumption and 5 points for rarely or never consumption. Detailed information regarding food grouping and assignment can be found in Table S1. In this study, PDI, hPDI, and uPDI were further stratified into three groups (T1, T2, and T3) based on the tertiles of the subjects’ scores.

### 2.3. Assessment of Frailty

In adherence to the frailty index (FI) construction standard [34], this study employed 35 health-related variables to formulate the FI. These variables encompassed self-rated health, psychological status, activities of daily living, sensory status, physical limitations, and chronic diseases (Table S2). Health status was assigned a score ranging from 0 to 1, contingent on the participant’s response to the respective variable. For instance, self-rated health status was categorised as healthy, moderately healthy, fair, unhealthy, and very unhealthy, with scores of 0, 0.25, 0.5, 0.75, and 1, respectively. Notably, 2 points were assigned if the subject had experienced two or more severe illnesses in the preceding two years. The FI was computed by dividing the aggregate health score by the total number of variables. In instances where study subjects had missing information on certain variables, deductions were applied in both the numerator and denominator. However, if the count of missing variables exceeded 30%, it was categorised as missing FI. In line with established literature [9,34,35], the FI was categorised into three scales: non-frail (FI ≤ 0.10), pre-frail (0.10 < FI ≤ 0.21), and frail (FI > 0.21). For our study, either prefrailty or frailty at the conclusion of the follow-up period was considered as the outcome of interest.

### 2.4. Assessment of Covariates

In consideration of factors identified in prior studies as potential influences on frailty, the covariates encompassed age (years), sex (male and female), type of residence (urban and rural), economic status (wealthy or not wealthy), living arrangement (solitary or not living alone), marital status (married and cohabiting or other), smoking status (current, former, or never), alcohol consumption (current, former, or never), exercise status (current, former, or never), and body-mass index (BMI; underweight, normal, overweight, or obese). The subjects’ height and weight were measured using standard methods and employed to calculate BMI = weight (kg)/height (m)^2^.

### 2.5. Statistical Analysis

Descriptive statistics were employed to calculate baseline characteristics. Cox proportional hazard models were constructed to scrutinize the association between the three plant-based diet indices and frailty in older adults, utilizing Schoenfeld residuals to assess the proportional risk hypothesis. Participant follow up was computed from baseline until the initial occurrence of the outcome of interest, death, loss to follow up, or the conclusion of the follow-up period, whichever transpired first. Restricted cubic spline curves, with 4 nodes chosen at the 5th, 35th, 65th, and 95th percentiles, were employed to explore potential nonlinear associations of baseline PDI, hPDI and uPDI with the frailty index. The models integrated multiple covariates to adjust for potential confounding factors affecting study outcomes. Model 1 incorporated adjustments for age and sex, while Model 2 additionally adjusted for type of residence, economic situation, residence status, marital status, smoking status, alcohol consumption, exercise status, and body mass index (BMI) based on Model 1. Subgroup analyses were conducted based on Model 2 to investigate whether the associations between plant-based diet indices and frailty exhibited variations by gender. The statistical analysis of this study was conducted using IBM SPSS version 20.0 and R version 4.2.1. GraphPad version 8.3 was utilised for visualising the results of subgroup analyses. A two-sided test with *p* < 0.05 was deemed statistically significant.

## 3. Results

### 3.1. Basic Information

A total of 2883 subjects, with an average age of 81 years, were included in this study, comprising 1331 females (46.2%) and 1552 males (53.8%). At the conclusion of the follow-up period, 1987 participants (68.9%) were either frail or pre-frail. The results indicated differences in PDI, hPDI, and uPDI scores at baseline among older adults by age, genders, marital statuses, smoking statuses, and BMI. There were differences in PDI and uPDI scores among individuals residing in different locations. Nevertheless, in different economic situations, residence statuses, alcohol consumption, and exercise statuses of the elderly, uPDI significant differences in scores. (*p* < 0.05) (Table 1). In addition, we compared the basic characteristics of participants of different genders, and there were statistically significant differences between males and females in age, residential status, marital status, smoking status, alcohol consumption status, exercise status, and BMI among older adults (*p* < 0.001) (Table S3).

### 3.2. Association between Baseline Plant-Based Diet Index and Frailty Index

**Figure 2:**
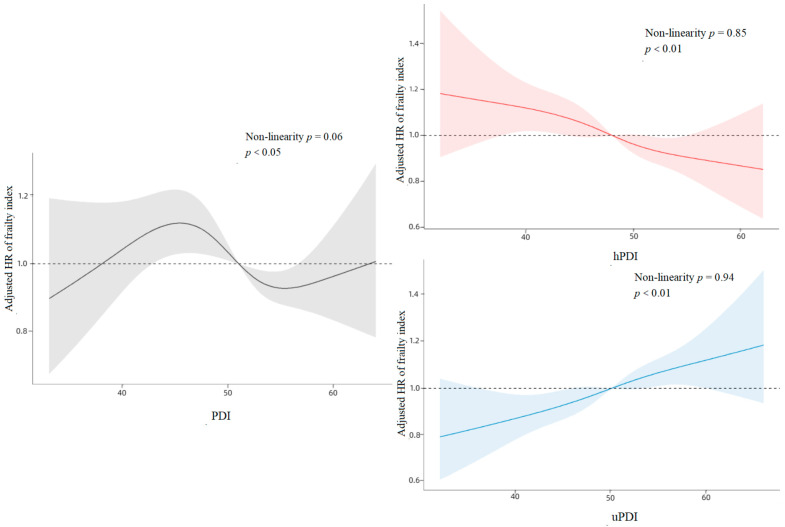
Cubic Spline Curves for PDI, hPDI, and uPDI Versus Association with Frailty.

The outcomes of the restricted cubic spline curves demonstrated statistically significant correlations (*p* < 0.05) of PDI, hPDI, and uPDI with the frailty index among older participants. Moreover, the correlations of PDI, hPDI, and uPDI with the frailty index exhibited linear tendencies (Figure 2). Upon stratifying the three plant-based diet indices into three groups based on participants’ scores’ tertiles, the highest tertile of PDI scores correlated with a 14% lower risk of frailty compared to participants in the lowest tertile of PDI in the fully adjusted model (HR = 0.86, 95% CI: 0.77–0.95, *p* < 0.01). Likewise, participants with the highest hPDI scores exhibited a 17% lower risk of frailty compared to those in the lowest hPDI tertile (HR = 0.83, 95% CI: 0.74–0.93, *p* < 0.01). Conversely, the highest quartile of uPDI scores correlated with a 21% increased risk of frailty compared to participants in the lowest quartile of uPDI scores (HR = 1.21, 95% CI: 1.08–1.36, *p* < 0.01) (Table 2).

### 3.3. Gender Differences in the Association between Plant-Based Diet Index and Frailty Index

**Figure 3:**
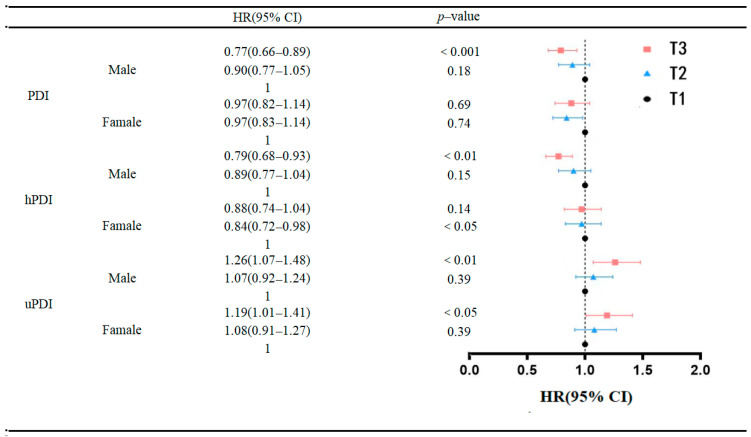
Association of baseline PDI, hPDI and uPDI with incidence of frailty risk.

To further investigate potential gender differences in the association between PDI, hPDI, uPDI and the onset of frailty in older adults, subgroup analyses were conducted based on gender subgroups. The results (Figure 3) demonstrated that the association between the highest tertile of PDI (HR = 0.77, 95% CI: 0.66–0.89, *p* < 0.001) and hPDI (HR = 0.79, 95% CI: 0.68–0.93, *p* < 0.01) scores and a reduced risk of frailty in males, compared to study participants in the lowest tertile of PDI and hPDI, was statistically significant. However, this association was not significant in females. Conversely, the association between the highest quartile of uPDI scores and an increased risk of developing frailty was statistically significant in both men (HR = 1.26, 95% CI: 1.07–1.48, *p* < 0.01) and women (HR = 1.19, 95% CI: 1.01–1.41) when compared to study participants in the lowest quartile of uPDI scores. Nevertheless, the detrimental effect was more pronounced in the male population than in the female population.

## 4. Discussion

The outcomes of the investigation revealed a correlation between overall plant-based diet index, healthful plant-based diet index (hPDI), and unhealthful plant-based diet index (uPDI) with the propensity for developing frailty. The outcomes from the restricted cubic spline analysis indicated that the consumption of a diet rich in plant-based constituents and low in animal-derived components exhibited an inversely proportional relationship with the likelihood of frailty onset in the elderly. Conversely, an increased risk of frailty was evident with elevated consumption of animal-based foods and diminished intake of plant-based foods in the geriatric population. Notably, PDI and hPDI exhibited a negative correlation with the susceptibility to frailty in the elderly, while uPDI demonstrated a positive association with frailty risk in this demographic. Furthermore, heightened adherence to PDI and hPDI exerted a more pronounced protective effect against frailty in males compared to females. Conversely, greater adherence to uPDI was notably linked to an increased risk of frailty in males. These findings suggest that the rationalization of dietary habits may function as a mitigating factor against frailty in the elderly.

Our findings indicate that higher PDI and hPDI are associated with a decreased risk of frailty in older adults. The role of a balanced diet in safeguarding the physical and mental health of a population has been extensively discussed [36,37,38,39]. Nonetheless, few studies have specifically evaluated the connection between the quality of a plant-based diet and the risk of frailty. Unlike specific diseases, frailty is often accompanied by a decline in the functioning of multiple physiological systems. Despite its prevalence increasing with age, frailty is an extreme consequence of normal aging. A previous cohort study with female nurses demonstrated that a higher adherence to a healthful plant-based diet was linked to a lower risk of frailty, while an unhealthful plant-based diet was associated with an increased risk [24]. Additionally, a study focused on older Brazilian and Italian women observed that older Brazilian women with a higher plant protein intake exhibited better physical functioning [40]. Similarly, a study based on a Chinese cohort found a significant negative association between high PDI and the risk of frailty in older adults [26]. However, contrasting findings exist. Another Chinese cohort study suggested that a dietary pattern including eggs, fish, and meat might reduce the incidence of frailty in older adults [41]. A cross-sectional study from Brazil indicated that relatively low meat consumption was linked to a high prevalence of frailty in edentulous individuals [42]. Hence, exploring the association between specific food groups and frailty warrants further investigation. Our study also revealed that a higher adherence to uPDI was associated with an increased risk of frailty in older adults. A U.S.-based cohort study noted that habitual consumption of unprocessed or processed red meat was associated with a higher risk of frailty [17]. Similarly, a study among community-dwelling older adults in Spain found that uPDI was associated with a higher risk of frailty, while the opposite was true for hPDI [25]. Some findings suggest that the consumption of ultra-processed foods, including dairy and meat products, strongly correlates with the risk of frailty in older adults. These findings emphasize the critical role of consuming unprocessed or minimally processed foods, such as vegetables and fruits, in preventing age-related frailty [43]. Interestingly, our results suggest that the protective effect of hPDI against frailty appears to be more pronounced in older adults compared to PDI. This implies that a reduced intake of refined grains, preserved vegetables, and sugar may be associated with a further reduction in the risk of frailty. However, the cause of this discrepancy, particularly whether it is related to the low intake of unhealthful plant-based foods, warrants further discussion. In future clinical practice, controlled clinical interventions around the role of specific food types in frailty related health disorders can be appropriately conducted.

Several pathophysiological mechanisms may help elucidate our findings. Firstly, inflammation and oxidative stress may contribute to frailty by inducing dysregulation in the organism. Plant-based foods, rich in flavonoids and antioxidants with anti-inflammatory and antioxidant properties, may mitigate the risk of frailty [44]. Secondly, imbalances in gut ecology can lead to immune responses and low-grade inflammation. Plant-based foods have been associated with greater gut microbiota diversity in older adults, potentially reducing inflammation and oxidative stress [45]. Additionally, insulin resistance (IR) increases as an individual ages [46]. Insulin resistance can cause frailty related health deficits by causing problems such as altered lipid metabolism, increased inflammatory status, impaired endothelial function, pro-thrombotic status, and atherosclerosis [47,48,49]. Moreover, deficiencies in macronutrients, such as protein, and micronutrients, such as vitamins, zinc and magnesium, negatively affect anabolic responses, levels of oxidative stress and levels of inflammation in the body in the elderly, leading to loss of individual muscle mass and tissue damage, thereby increasing the risk of frailty in the elderly [50,51,52,53,54]. Vegetable, fruit and other plant foods are often rich in macronutrients and micronutrients. Finally, a high consumption of animal diets, including meat, sugar, and dairy products, is often associated with unfavourable levels of low-grade inflammation and glucose metabolic biomarkers, elevated levels of oxidative stress, and metabolic disorders in the body, which can lead to chronic diseases such as type 2 diabetes and cardiovascular disease [55,56]. This is closely related to the pathogenesis of frailty.

Significantly, upon stratifying participants by gender, our findings demonstrated that although the association of the plant-based diet index with the development of frailty was statistically significant for both men and women, higher adherence to PDI and hPDI was more protective against developing frailty in men compared to women. Simultaneously, higher adherence to uPDI was also more significantly detrimental to frailty in men. This gender-specific difference may be attributed to biological, psychological, social, and behavioural distinctions between women and men. Women typically bear a higher burden of chronic disease and more severe disability [57], exhibit lower levels of physical activity [58], are more susceptible to mood disorders and stress [59], and often lack social support [60,61], among other factors contributing to a higher risk of frailty in females [62]. This could diminish the impact of the plant-based diet compared to the animal-based diet on frailty risk in females. Additionally, it has been noted that the relevance of diet in the pathogenesis of female frailty is lower than that of males if the body is already in an inflammatory state due to the influence of other factors [58]. Furthermore, due to differences between sex chromosomes, males exhibit higher innate and pro-inflammatory activity, rendering them more susceptible to inflammatory diets than females [63]. Therefore, based on the multidimensional nature of frailty, the progress of frailty can be interfered in multiple areas such as diet, physical activity, mental health and drug optimization. In addition, since the role of diet can play a more important role in the process of male frailty, it can be effectively prevented and delayed by forming a healthy diet pattern of hypoinsulinemia, low inflammation and reducing the risk of diabetes, providing the necessary macronutrients and micronutrients to ensure body function and maintain homeostasis, thereby effectively preventing and delaying frailty [58,64].

The present study boasts several strengths. Unlike other investigations relying on simple indicators, our study comprehensively assesses frailty status using Frailty Indices (FIs) that encompass all major health domains. Furthermore, the study utilizes nationally representative data with an extended follow-up period. However, certain limitations should be acknowledged. Firstly, participants’ dietary information was obtained through interviews, introducing potential recall bias. Secondly, the questionnaire, focusing solely on the frequency of food intake and not addressing the amount consumed, precluded the calculation and adjustment of total energy intake. Thirdly, despite extensive adjustment for confounders, the potential for confounding bias persists. Fourth, the classification of indicators used to construct the plant-based diet indices and frailty index is worth exploring in a more rigorous way to highlight different risk groups. Fifth, this study had a long follow-up period, and this study used baseline dietary information from study subjects to calculate the plant-based diet indices for older adults, which may have overlooked possible changes in participants’ dietary patterns. Lastly, the study’s primary focus on the Chinese elderly population restricts the generalizability of findings to other ethnic and racial populations.

Older people have limited access to ideal dietary patterns, and the burden of health defects attributed to irrational diets can be very high, especially among older men. Therefore, in the future social policy practice, the diet pattern based on healthy plant food or other healthy diet guidance patterns can be promoted in communities or hospitals, and health education can be carried out among the elderly to change the concept of healthy diet for the elderly. Different interventions should be taken for frail elderly people with different dietary habits, and the role of primary health care should be played to enhance the sustainability of healthy poverty reduction from the source.

## 5. Conclusions

In conclusion, the current study establishes an association between high adherence to PDI and hPDI and a reduced risk of frailty in older adults. Conversely, high adherence to uPDI is associated with an increased risk of frailty in older adults. Furthermore, the protective effects of PDI and hPDI against frailty, as well as the detrimental effects of uPDI, exhibit more pronounced trends in men compared to women. The findings underscore the pivotal role of appropriate dietary behaviours in influencing frailty risk among older adults. This implies that judicious control of plant-based food intake and reduction of animal-based food intake can contribute to preventing the onset and managing the progression of frailty, particularly in the male elderly population.

## Figures and Tables

**Table 1:** Baseline characteristics of participants.

Characteristics	N(%)	PDI			*p*-Value	hPDI			*p*-Value	uPDI			*p*-Value
		T1	T2	T3		T1	T2	T3		T1	T2	T3	
Age (years)	2883 (100)	82 (11)	81 (11)	79 (10)	<0.001	83 (11)	80 (11)	80 (10)	<0.001	80 (11)	81 (11)	82 (11)	<0.001
Sex					<0.05				<0.001				<0.001
Male	1552 (53.8)	562 (36.2)	469 (30.2)	521 (33.6)		479 (30.9)	567 (37.1)	497 (32.0)		622 (40.1)	525 (33.8)	405 (26.1)	
Female	1331 (46.2)	506 (38.0)	439 (33.0)	386 (29.0)		486 (36.5)	508 (38.2)	337 (25.3)		403 (30.3)	497 (37.3)	431 (32.4)	
Resdic					<0.01				0.09				<0.001
Urban	361 (12.5)	122 (33.8)	142 (39.3)	97 (26.9)		103 (28.5)	142 (39.3)	116 (32.1)		235 (65.1)	78 (21.6)	48 (13.3)	
Town	2522 (87.5)	946 (37.5)	766 (30.4)	810 (32.1)		862 (34.2)	942 (37.4)	718 (28.5)		790 (31.3)	944 (37.4)	788 (31.2)	
Economic situation					0.23				0.31				<0.001
Wealthy	483 (16.8)	189 (39.1)	158 (32.7)	136 (28.2)		162 (33.5)	169 (35.0)	152 (31.5)		257 (53.2)	145 (30.0)	81 (16.8)	
Not wealthy	2400 (83.2)	879 (36.6)	750 (31.2)	771 (32.1)		803 (33.5)	915 (38.1)	682 (28.4)		768 (32.0)	877 (36.5)	755 (31.5)	
Cohabitation status					0.15				0.62				<0.001
Solitude	544 (18.9)	220 (40.4)	168 (30.9)	156 (28.7)		190 (34.9)	205 (37.7)	149 (27.4)		140 (25.7)	189 (34.7)	215 (39.5)	
Not living alone	2339 (81.1)	848 (36.3)	740 (31.6)	751 (32.1)		775 (33.1)	879 (37.6)	685 (29.3)		885 (37.8)	833 (35.6)	621 (26.5)	
Marital status					<0.001				<0.001				<0.001
Married/cohabitating	1306 (45.3)	415 (31.8)	400 (30.6)	491 (37.6)		363 (27.8)	508 (38.9)	435 (33.3)		539 (41.3)	451 (34.5)	316 (24.2)	
Others	1577 (54.7)	653 (41.4)	508 (32.2)	416 (26.4)		602 (38.2)	576 (36.5)	399 (25.3)		486 (30.8)	571 (36.2)	520 (33.0)	
Smoking status					<0.01				<0.01				<0.01
never	1718 (59.6)	675 (39.3)	542 (31.5)	501 (29.2)		613 (35.7)	630 (36.7)	475 (27.6)		576 (33.5)	608 (35.4)	534 (31.1)	
former	429 (14.9)	153 (35.7)	134 (31.2)	142 (33.1)		132 (30.8)	180 (42.0)	117 (27.3)		177 (71.3)	151 (35.2)	101 (23.5)	
now	736 (25.5)	240 (32.6)	232 (31.5)	264 (35.9)		220 (29.9)	274 (37.2)	242 (32.9)		272 (37.0)	263 (35.7)	201 (27.3)	
Alcohol consumption					0.53				0.12				<0.001
never	1813 (62.9)	687 (37.9)	573 (31.6)	553 (30.5)		619 (34.1)	694 (38.3)	500 (27.6)		598 (33.0)	646 (35.6)	569 (31.4)	
former	357 (12.4)	124 (34.7)	118 (33.1)	115 (32.2)		126 (35.3)	118 (33.1)	113 (31.7)		134 (37.5)	122 (34.2)	101 (28.3)	
now	713 (24.7)	257 (36.0)	217 (30.4)	239 (33.5)		220 (30.9)	272 (38.1)	221 (31.0)		293 (41.1)	254 (35.6)	166 (23.3)	
Physical exercise					0.18				0.09				<0.001
never	1611 (55.9)	622 (38.6)	494 (30.7)	495 (30.7)		569 (35.3)	602 (37.4)	440 (27.3)		470 (29.2)	618 (38.4)	523 (32.5)	
former	255 (8.8)	97 (38.0)	73 (28.6)	85 (33.3)		73 (28.6)	99 (38.8)	83 (32.5)		90 (35.3)	92 (36.1)	73 (28.6)	
now	1017 (35.3)	349 (34.3)	341 (333.5)	327 (32.2)		323 (31.8)	383 (37.7)	311 (30.6)		465 (45.7)	312 (30.7)	240 (23.6)	
BMI (kg/m^2^)					<0.001				<0.001				
low	696 (24.1)	317 (45.5)	214 (30.7)	165 (23.7)		283 (40.7)	238 (34.2)	175 (25.1)		212 (30.5)	258 (37.1)	226 (32.5)	<0.001
nomal	1749 (60.7)	619 (35.4)	549 (31.4)	581 (33.2)		575 (32.9)	664 (38.0)	510 (29.2)		610 (34.9)	624 (35.7)	515 (29.4)	
overweight	355 (12.3)	112 (31.5)	116 (32.7)	127 (35.8)		89 (25.1)	146 (41.1)	120 (33.8)		157 (44.2)	120 (33.8)	78 (22.0)	
Obesity	83 (2.9)	20 (24.1)	29 (34.9)	34 (41.0)		18 (21.7)	36 (43.4)	29 (34.9)		46 (55.4)	20 (24.1)	17 (20.5)	

PDI—overall plant-based diet index; hPDI—healthful plant-based diet index; uPDI—unhealthful plant-based diet index; BMI—body-mass index.

**Table 2:** Association of Baseline PDI, hPDI and uPDI with Incidence of Frailty Risk.

	T1	T2		T3	
		HR (95% CI)	*p*-Value	HR (95% CI)	*p*-Value
PDI					
Model 1	1	0.93 (0.84–1.04)	0.21	0.85 (0.77–0.95)	<0.01
Model 2	1	0.93 (0.83–1.04)	0.19	0.86 (0.77–0.95)	<0.01
hPDI					
Model 1	1	0.87 (0.79–0.97)	<0.05	0.82 (0.73–0.92)	<0.001
Model 2	1	0.87 (0.79–0.97)	<0.05	0.83 (0.74–0.93)	<0.001
uPDI					
Model 1	1	1.07 (0.96–1.19)	0.21	1.23 (1.10–1.38)	<0.001
Model 2	1	1.05 (0.94–1.18)	0.37	1.21 (1.08–1.36)	<0.01

Note: OR—odds ratio; CI—confidence interval. Grouping basis of three plant-based diet indices: PDI (T1 ≤ 48, 49 ≤ T2 ≤ 53, T3 ≥ 54); hPDI (T1 ≤ 45, 46 ≤ T2 ≤ 50, T3 ≥ 51); uPDI (T1 ≤ 47, 48 ≤ T2 ≤ 53, T3 ≥ 54). Model 1 adjusted for age and gender; Model 2 adjusted for type of residence, economic situation, residence status, marital status, smoking status, alcohol consumption, exercise status, and BMI based on Model 1.

## Data Availability

Data are contained within the article and Supplementary Materials.

## Supplementary Materials

The following supporting information can be downloaded at: https://www.mdpi.com/article/10.3390/nu15245120/s1, Table S1. Scoring of plant-based diet indices. Table S2. Scoring of frailty index. Table S3: Baseline characteristics of participants by sex.
