# Supplementary material for: Plant-Based Diet Indices and Their Association with Frailty in Older Adults: A CLHLS-Based Cohort Study
Source: Nutrients. 2023 Dec 15;15(24):5120. doi: 10.3390/nu15245120 (PMC10745508; doi:10.3390/nu15245120)
Supplement: Supplementary file 1 [file nutrients-15-05120-s001.zip › nutrients-2751936-supplementary.pdf]

Contents

Table S1. Scoring of plant-based diet indices. 1
Table S2. Scoring of frailty index...3
Table S3. Baseline characteristics of participants by sex...5

Table S1. Scoring of plant-based diet indices.

Types	Food	Frequency	PDI	hPDI	uPDI
Healthful plant-based food	Whole grain	Yes	5	5	1
		No	1	1	5
	Vegetable oil	Yes	5	5	1
		No	1	1	5
	Fresh fruit	Almost everyday	5	5	1
		Except winter	4	4	2
		Occasionally	2	2	4
		Rarely or never	1	1	5
	Vegetable	Almost everyday	5	5	1
		Except winter	4	4	2
		Occasionally	2	2	4
		Rarely or never	1	1	5
	Legume	Almost everyday	5	5	1
		≥1 time/week	4	4	2
		≥1 time/month	3	3	3
		Occasionally	2	2	4
		Rarely or never	1	1	5
	Garlic	Almost everyday	5	5	1
		≥1 time/week	4	4	2
		≥1 time/month	3	3	3
		Occasionally	2	2	4
		Rarely or never	1	1	5
	Nut products	Almost everyday	5	5	1
		≥1 time/week	4	4	2
		≥1 time/month	3	3	3
		Occasionally	2	2	4
		Rarely or never	1	1	5
	Tea	Almost everyday	5	5	1
		≥1 time/week	4	4	2
		≥1 time/month	3	3	3
		Occasionally	2	2	4
		Rarely or never	1	1	5
Unhealthful plant-based food	Refined grain	Yes	5	1	5
		No	1	5	1
	Salt-preserved vegetable	Almost everyday	5	1	5
		≥1 time/week	4	2	4
		≥1 time/month	3	3	3
		Occasionally	2	4	2
		Rarely or never	1	5	1
	Sugar	Almost everyday	5	1	5
		≥1 time/week	4	2	4
		≥1 time/month	3	3	3
		Occasionally	2	4	2
		Rarely or never	1	5	1

Animal-based food	Animal fat	Yes	1
		No	5
	Meat	Almost everyday	1
		≥1 time/week	2
		≥1 time/month	3
		Occasionally	4
		Rarely or never	5
	Fish	Almost everyday	1
		≥1 time/week	2
		≥1 time/month	3
		Occasionally	4
		Rarely or never	5
	Egg	Almost everyday	1
		≥1 time/week	2
		≥1 time/month	3
		Occasionally	4
		Rarely or never	5
	Milk products	Almost everyday	1
		≥1 time/week	2
		≥1 time/month	3
		Occasionally	4
		Rarely or never	5

Table S2. Scoring of frailty index.

	Variables	Values
**Basic situation**	1. self-reported health	very good=0; good=0.25; so so=0.5; bad=0.75; very bad=1
	2. feel fearful or anxious	never=0; seldom=0.25; sometimes=0.5; often=0.75; always=1;
	3. feel useless with age	never=0; seldom=0.25; sometimes=0.5; often=0.75; always=1;
	4. look on the bright side of things	always=0; often=0.25; sometimes=0.5; seldom=0.75; never =1;
	5. keep my belongings neat and clean	always=0; often=0.25; sometimes=0.5; seldom=0.75; never =1;
	6. make own decision	always=0; often=0.25; sometimes=0.5; seldom=0.75; never =1;
	7. the health of interviewee rated by interviewer	surprisingly healthy=0; relatively healthy=0.33; moderately ill=0.67; very ill=1
**Basic ability**	8. bathing	without assistance=0; one part assistance=0.5; more than one part assistance=1
	9. dressing	without assistance=0; one part assistance=0.5; more than one part assistance=1
	10. toileting	without assistance=0; some assistance=0.5; don't use toilet=1
	11. indoor transferring	without assistance=0; with assistance=0.5; bedridden=1
	12. continence	without assistance=0; occasional accidents=0.5; incontinent=1
	13. feeding	without assistance=0; with some help=0.5; need feeding=1
	14. do you do house work at present?	almost everyday=0; ≥1/ week=0.25; ≥1/month& < 1/week=0.5; < 1/month=0.75; never=1
	15. visual function	can see and distinguish=0; can see only=0.33; can't see=0.67; blind=1
	16. was interviewee able to hear?	yes, without hearing aid=0; yes, but needs hearing aid=0.33; partly, despite hearing aid=0.67; no=1
	17. heart rate	< 80=0; ≥80=1
	18. hand behind neck	both hands=0; right hand=0.5; left hand=0.5; neither hand=1
	19. hand behind lower back	both hands=0; right hand=0.5; left

		hand=0.5; neither hand=1
	20. able to stand up from sitting in a chair?	yes, without using hands=0; yes, using hands=0.5; no=1
	21. able to pick up a book from the floor?	yes, standing=0; yes, sitting=0.5; no=1
	22. able to use chopsticks to eat?	yes=0; no=1
	23. steps used to turn around 360 with help?	≤4=0; >4=0.5; cannot turn around=1
**Medical history**	24. Number of times suffering from serious illness within the past two years	0=0; 1=1; >1=2
	25. suffering from hypertension?	no=0; yes=1
	26. suffering from diabetes?	no=0; yes=1
	27. suffering from diabetes?	no=0; yes=1
	28. suffering from stroke or cvd?	no=0; yes=1
	29. suffering from bronchitis, emphysema, pneumonia, asthma?	no=0; yes=1
	30. suffering from tuberculosis?	no=0; yes=1
	31. suffering from cancer?	no=0; yes=1
	32. suffering from gastric or duodenal ulcer?	no=0; yes=1
	33. suffering from parkinson's disease?	no=0; yes=1
	34. suffering from bedsore?	no=0; yes=1
	35. suffering from dementia?	no=0; yes=1

Table S3. Baseline characteristics of participants by sex.

Characteristics	N	Sex		χ^2	*P*
		Male	Female		
Age(years)	2883(100)	80(10)	82(11)	24.30	< 0.001
Resdic				0.89	0.35
Urban	361(12.5)	186(51.5)	175(48.5)		
Town	2522(87.5)	1366(54.2)	1156(45.8)		
Economic situation				1.96	0.18
Wealthy	483(16.8)	274(56.7)	209(43.3)		
Not wealthy	2400(83.2)	1278(53.2)	1122(46.8)		
Cohabitation status				15.97	< 0.001
Solitude	544(18.9)	251(46.1)	293(53.9)		
Not living alone	2339(81.1)	1301(55.6)	1038(44.4)		
Marital status				222.92	< 0.001
Married/cohabitating	1306(45.3)	902(69.1)	404(30.9)		
Others	1577(54.7)	650(41.2)	927(58.8)		
Smoking status				899.56	< 0.001
never	1718(59.6)	531(30.9)	1187(69.1)		
former	429(14.9)	370(86.2)	59(13.8)		
now	736(25.5)	651(88.5)	85(11.5)		
Alcohol consumption				518.61	< 0.001
never	1813(62.9)	682(37.6)	1131(62.4)		
former	357(12.4)	280(78.4)	77(21.6)		
now	713(24.7)	590(82.7)	123(17.3)		
Physical exercise				37.84	< 0.001
never	1611(55.9)	787(48.9)	824(51.1)		
former	255(8.8)	145(56.9)	110(43.1)		
now	1017(35.3)	620(61.0)	397(39.0)		
BMI(kg/m2)				37.61	< 0.001
low	696(24.1)	313(45.0)	383(55.0)		
nomal	1749(60.7)	1004(57.4)	745(42.6)		
overweight	355(12.3)	201(56.6)	154(43.4)		
Obesity	83(2.9)	34(41.0)	49(59.0)		
